# Is there an alternative explanation to post-MI emergence of mitral regurgitation; a CMR-LGE observational study

**DOI:** 10.1186/1532-429X-14-S1-O79

**Published:** 2012-02-01

**Authors:** Hari Bogabathina, Mark Doyle, Ronald B Williams, June Yamrozik, Diane A Vido, Robert W Biederman

**Affiliations:** 1 Allegheny General Hospital, Pittsburgh, USA

## Summary

LGE of the mitral valve and annulus may explain emergence of post-MI mitral regurgitation.

## Background

Multiple factors contribute to the mechanisms for post-MI mitral regurgitation. It is postulated that a passive process primarily drives remodeling of adjacent myocardium. However, non-geometric, i.e. active mechanisms have not been considered as potential contributors to mitral valve pathology. Standard CMR late gadolinium enhancement (LGE) delineates multiple LV myocardial histopathologies and may also be sensitive to non-myocardial pathology involving the mitral valve leaflets.

We hypothesize that LGE may detect a unique reactive process of the mitral valve; MVE (mitral valve enhancement) in post-MI patients and the incidence of MR may correlate with the presence of MVE.

## Methods

Presence or absence of MVE was noted in pts presenting for CMR with MI and non-MI indications requiring LGE. MR was semi-quantitatively graded on a scale of 0-4+. Chi square analysis was performed for non-contiguous variables.

## Results

Patients (87; M=54, F=33) underwent 115 LGE-CMR studies (1.5T GE, Milwaukee, WI) with 0.2mmol/kg of Magnevist (Berlex, Wayne, NJ) or 0.1mmol/kg MultiHance (Bracco, Princeton, NJ). LGE was noted in 95 and no LGE was noted in 20 studies. Post-MI patterns of LGE were present in 73 pts and absent in 42. MVE+ (present) in 54, whereas MVE- (absent) in 61. MR was present in 89 and MR was absent in 26 studies. The MVE+ phenomenon was observed chiefly in post-MI pts (47 of 73; 63%) and infrequently in non-post-MI pts (7 of 42; 17%); χ2 = 22, df=1, p<0.001. Further, in post-MI pts with MR, MVE was much more frequent (42 vs. 22) whereas in non-post MI with MR, MVE was less frequent (5 vs. 20); χ2 = 13, df=1, p<0.001.

## Conclusions

LGE-CMR depicts mitral valve enhancement in a large number of post-MI patients but rarely in non-post-MI patients. Post-MI patients with MVE were far more likely to have MR than patients without MVE. These observations suggest a specific, as yet unkown reactive process may contribute to mitral leaflet structural deterioration in post-MI patients. This active phenomenon was not a suspected contributor to the post-MI MR phenotype but, via CMR, appears to be important in the formation of post-MI mitral regurgitation. These observations suggest a specific, as yet unkown reactive process may contribute to mitral leaflet structural deterioration in post-MI patients

## Funding

Internal.

**Figure 1:**
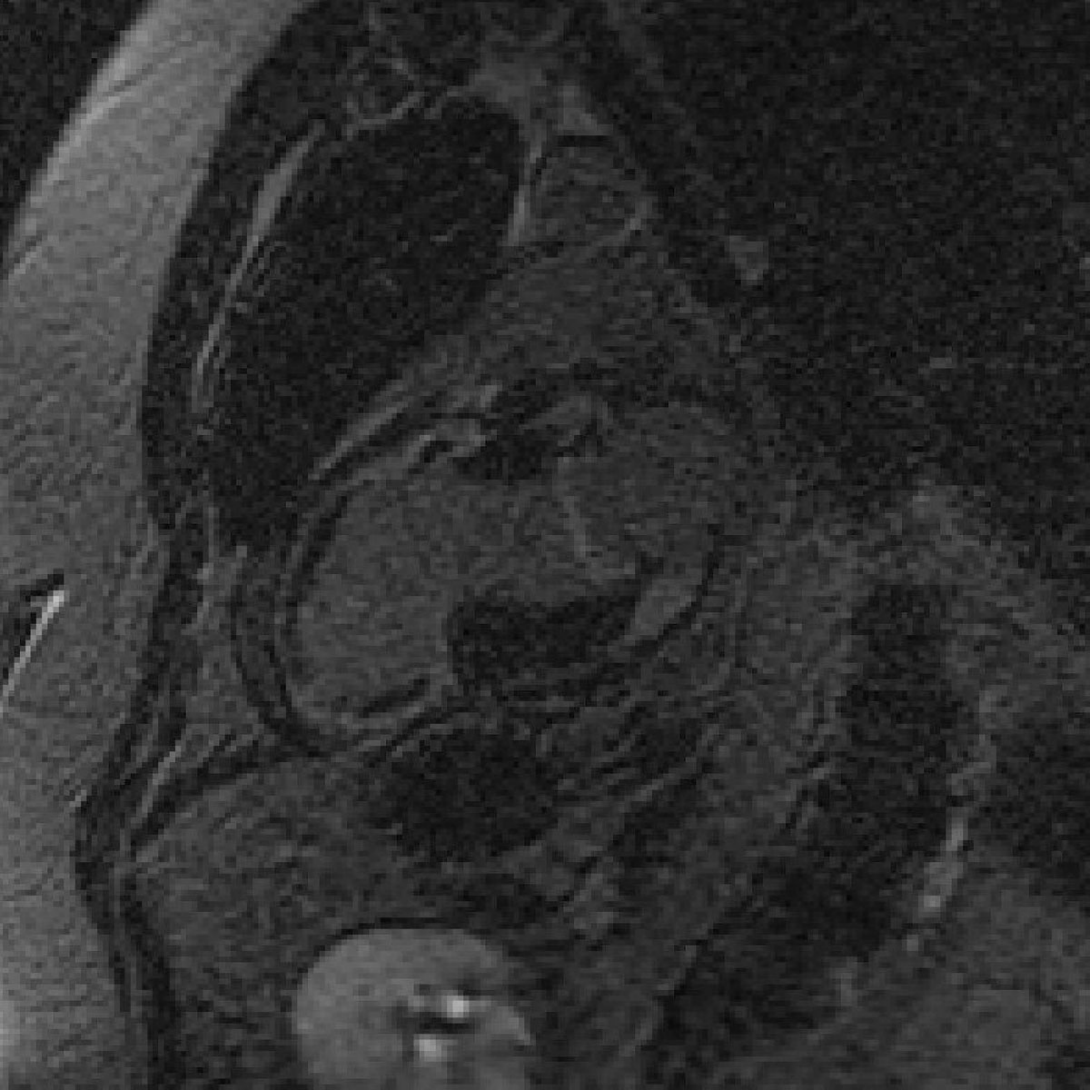
Concurrent Mitral valve and Myocardial Infarct enhancment 20min after Gadolinium administration.

